# Editorial: Pain assessment and management in veterinary medicine

**DOI:** 10.3389/fvets.2024.1549243

**Published:** 2025-01-17

**Authors:** Caterina Di Bella, Petra Dmitrović, Alessandro Mirra, Luca Pennasilico

**Affiliations:** ^1^School of Bioscience and Veterinary Medicine, University of Camerino, Camerino, Italy; ^2^Faculty of Veterinary Medicine, University of Zagreb, Zagreb, Croatia; ^3^Vetsuisse Faculty, University of Bern, Bern, Switzerland

**Keywords:** acute pain, chronic pain, pain diagnosis, pain treatment, veterinary medicine

Despite the complexity of identifying and treating painful states in companion animals, several studies have been conducted to explore the topic and provide knowledge to properly manage pain in the veterinary field (1). This Research Topic has collected 10 scientific articles related to the assessment and management of pain in different animal species.

Casas-Alvarado et al. evaluated the nociceptive response to CBD alone and in combination with meloxicam in dogs undergoing ovariohysterectomy, using the technique of infrared pupillometry. In this study, a double result was obtained: (1) the evaluation of nociception by infrared pupillometry could be of valuable aid in dogs affected by acute pain; (2) CBD provides good postoperative analgesia both alone and with the anti-inflammatory, representing a good therapeutic alternative. The pupillometry method for the evaluation of acute pain has also been studied in equine species by Mascaró Triedo et al. in order to evaluate the painful response to nose twitching in horses. In agreement with the previous study, the authors state that the subjects showed pupillary dilation following the manipulation described above. However, one aspect to consider is that the modification of pupil diameter could be influenced by the administration of analgesic drugs such as acepromazine, which promotes pupil dilation, or romifidine, which, instead, inhibits it. To date, there are different pain recognition methods and, among those most described in the literature, the Grimace scales stand out. Chiavaccini et al. published a narrative review that provides an overview of animal pain recognition technologies, starting from the classic Grimace Scales that, although valid, have limitations related to the subjectivity of the assessments and the need for basic operator training, up to the description of automatic pain recognition (APR) based on artificial intelligence. These technologies, thanks to the analysis of facial expressions, body language, vocalizations and physiological parameters, allow to obtain complete data on the patient's pain status and offer a promising progress in veterinary field.

One area of veterinary medicine where pain recognition and management are fundamental is animal experimentation. Ensuring good analgesia in experimental subjects is ethically essential (2). Petrucci et al. evaluated the usefulness of the Nociceptive Withdrawal Reflex (NWR) to assess antinociception following spinal analgesia (morphine and ropivacaine) in pigs undergoing veno-arterial extracorporeal membrane oxygenation (VA-ECMO). Nociceptive withdrawal reflex thresholds increased significantly following spinal injection, and an effect was seen on average up to 6 h. This study supports the usefulness of the NWR for evaluating antinociception following spinal analgesia in experimental pigs, especially if the evaluation of cardiovascular variables are not reliable, as during VA-ECMO.

Equally complex is the assessment of chronic pain. In dogs, diagnostic capabilities have improved with the introduction of Owner-Reported Outcome Measures. Among these, the Liverpool Osteoarthritis in Dog (LOAD) is widely used. It is a questionnaire to be submitted to the owners of dogs with osteoarthritis (OA). One limitation is that LOAD is distributed in English. The aim of the study by Olcoz et al. was to develop a version of the LOAD in Spanish, equivalent to the English one. This scientific production promotes the use of the LOAD by Spanish-speaking veterinarians, researchers and owners, improving the assessment of chronic OA pain in dogs.

Regarding the treatment of acute and chronic pain, new research has been introduced in both small and large animals with the common goal of improving the quality of life of our pets. Wickstead et al., retrospectively analyzed the incidence of complications related to the application of a wound infusion catheter (WIC) in horses undergoing partial ostectomy of the thoracolumbar vertebral processes, with the aim of documenting whether the presence of the catheter could induce negative events in the post-operative period. The extrapolated results confirm the absence of correlation between the application of the WIC and the development of infections and secondary complications. Based on this, the authors encourage further scientific research on the topic as it could represent a valid tool for the management of pain induced by spinal surgery in equine species. A study on the application of loco-regional analgesia in large animals was also carried out by Interlandi et al., who evaluated the efficacy of the local application of butorphanol alone and in association with lidocaine in calves undergoing umbilical hernia repair. The study demonstrated that both protocols are safe and valid in ensuring good intra- and post-operative analgesia in this species, reducing pain and stress, and increasing animal welfare.

Pain treatments in dogs are largely studied, especially in the field of orthopedic pain. Galosi et al., evaluated the synergistic efficacy of Magnesium Sulfate (MgSO_4_) and Ketamine in dogs undergoing TPLO. The aim was to evaluate whether MgSO_4_, acting in potentiating synergism with Ketamine, could provide better perioperative analgesia. The results showed that the ketamine/MgSO_4_ association resulted in a lower rescue analgesia request and opioid consumption. This protocol could represent a useful analgesic support in dogs affected by orthopedic acute pain.

Instead, regarding the management of chronic pain from orthopedic pathology, such as osteoarthritis, different analgesic protocols have been studied in dogs. A valid example is the study conducted by Enomoto et al., who evaluated the efficacy of the association grapiprant/fish oil/exercise in young dogs affected by OA, for a period of 4 months, demonstrating that the multimodal treatment guaranteed a significant clinical benefit to the treated subjects. Similarly, Kampa et al., compared the efficacy of green-lipped mussel/krill oil, meloxicam, *Biota orientalis* extracts and a placebo (sunflower oil) for 6 weeks in young dogs affected by OA. Results showed a significant clinical benefit in patients that received the anti-inflammatory or the green-lipped mussel/krill oil compared to the other two groups. Data obtained could be significant in defining a suitable multimodal therapy that includes the synergism between multiple molecules.

In conclusion, the results of studies and reviews mentioned include interesting findings on diagnosis and treatment of pain in small and large animals, that contribute to a constant improvement in our clinical and experimental activity.
